# Bempedoic acid and its role in contemporary management of hyperlipidemia in atherosclerosis

**DOI:** 10.1080/07853890.2022.2059559

**Published:** 2022-05-09

**Authors:** Ramyashree Tummala, Manasvi Gupta, Arvind Reddy Devanabanda, Dhrubajyoti Bandyopadhyay, Wilbert S. Aronow, Kausik K. Ray, Mamas Mamas, Raktim K. Ghosh

**Affiliations:** a Department of Cardiology, Mount Sinai Beth Israel, New York, NY, USA; b Department of Internal Medicine, University of Connecticut Health Center, Farmington, CT, USA; c Department of Cardiology, Donald and Barbara Zucker School of Medicine at Hofstra/Northwell, Manhasset, NY, USA; d Department of Cardiology, Westchester Medical Center and New York Medical College, New York, NY, USA; e Imperial Centre for Cardiovascular Disease Prevention, London, UK; f Keele Cardiac Research Group, Institutes of Science and Technology in Medicine and Primary Care Keele University, Stoke-on-Trent, UK; g MedStar Heart and Vascular Institute, Union Memorial Hospital, Baltimore, MD, USA

**Keywords:** Bempedoic acid, atherosclerosis, CLEAR HARMONY, CLEAR WISDOM, CLEAR OUTCOMES

## Abstract

Atherosclerotic heart disease is the leading cause of mortality and morbidity in the USA. Low density lipoprotein (LDL) has been the target for many hypolipidemic agents to modify atherosclerotic risk. Bempedoic acid is a novel hypolipidemic drug that inhibits the enzymatic activity of ATP citrate lyase in the cholesterol synthesis pathway. CLEAR Harmony, CLEAR Wisdom, CLEAR Tranquillity and CLEAR Serenity have shown safety and efficacy associated with long term administration of this drug. Studies have shown effectiveness in reducing LDL-C in both statin intolerant patients and in patients on maximally tolerated doses of statin. The fixed drug combination of bempedoic acid and ezetimibe in a recent phase III showed significant reduction in LDL compared with placebo, which might be a promising future for LDL reduction among statin intolerant patients. Bempedoic acid also reduced inflammatory markers like hs-CRP. Given these results, bempedoic acid alone and in combination with ezetimibe received the USA FDA approval for adults with heterozygous familial hypercholesterolaemia or established atherosclerotic cardiovascular disease. We present a comprehensive review exploring the underlying mechanism, pre-clinical studies, and clinical trials of bempedoic acid and discuss the potential future role of the drug in treating hyperlipidaemia.

## 1. Introduction

Cardiovascular disease (CVD) is the leading cause of death in the United States of America (USA) [1]. Hyperlipidaemia is a major modifiable risk factor for the development of atherosclerotic cardiovascular disease (ASCVD) and represents an important target for therapeutic interventions. LDL cholesterol is a well-known risk factor for ASCVD and has been the primary target for LDL lowering agents and lifestyle modifications. Statins have been the mainstay of medical therapy in hyperlipidaemia management for almost three decades but have adverse effects on medication compliance [2]. Furthermore, maximally tolerated doses of statins often fail to achieve the target LDL goal. While observational studies have indicated that about 10–15% of patients have statin intolerance, almost 1.5–5% of patients in randomized controlled trials have been noted to have statin intolerance [3]. The most recent American Heart Association (AHA)/American College of Cardiology (ACC)/Multi-societies 2018 cholesterol management guidelines recommended target LDL-C goal of less than 70 mg/dL in high-risk ASCVD patients [4]. The 2019 European Society of Cardiology (ESC)/European Atherosclerosis (EAS) guidelines suggest a lower target goal LDL-C less than 55 mg/dL for patients with very high-risk of ASCVD for primary and secondary prevention [5]. High intensity or maximally tolerated statin therapy often fails to achieve this lipid goal, thus necessitating the use of other non-statin hypolipidemic agents. Ezetimibe and proprotein convertase subtilin-kexin type 9 (PCSK9) inhibitors are recommended by the ACC/AHA/Multi-societies 2018 and ESC/EAS 2019 lipid guidelines to achieve the target LDL-C levels [4]. The use of ezetimibe along with statin therapy has been shown to cause an additional lowering of 13–20% in LDL-C levels [6]. There is an additional 43–64% of LDL-C reduction with the use of PCSK9 inhibitors along with statins [7–9]. PCSK9 inhibitors have not only been shown to reduce LDL-C level but also been shown to have significant mortality and cardiovascular morbidity benefits. However, there are major barriers in using PCSK9 inhibitors, including cost and subcutaneous administration [10].

Bempedoic acid is a new hypolipidemic drug with a novel mechanism of action that targets cholesterol synthesis through inhibition of the enzyme ATP citrate lyase. Bempedoic acid received USA Food and Drug Administration (FDA) approval in February 2020 for adults with heterozygous familial hypercholesterolaemia or established ASCVD [11]. The bempedoic acid and ezetimibe (Nexlizet) combination received USA FDA approval for the same indication [12]. We present a comprehensive review exploring the underlying mechanism, pre-clinical studies, and clinical trials of bempedoic acid with our opinion of the future therapeutic directions with this drug (Table 1).

**Table 1.** Pharmacology of bempedoic acid [12–19].

Bempedoic acid gets activated in liver to an active metabolite Activated drug inhibits the enzyme ATP Citrate lyase (ACLY) Oral dose, 180mg once daily Elimination half-life is 15–24 hours Initial trials showed LDL-C lowering efficacy of upto 27% as monotherapy Added LDL lowering of upto 48% with Bempedoic acid and ezetimibe combination Lower incidence of myopathy compared with statin

**Table 2.** Comparing pharmacology of novel non statin hypolipidemic drugs.

Drugs	Alirocumab (PCSK-9 inhibitor)	Evolocumab (PCSK-9 inhibitor)	Inclisiran	Bempedoic acid
Dose	75–150mg Bi-weekly or 300 mg monthly	140mg Bi-weekly or 420 mg monthly	100–150mg Every 6 months	180mg Once daily
Route	Subcutaneous	Subcutaneous	Subcutaneous	Oral
Metabolism	Binds to PCSK9 enzyme and proteolysis	Binds to PCSK9 enzyme and proteolysis	–	–
Elimination t_1/2_	11–20 days	11–20 days	–	15–24hrs
Effectiveness (%LDL-C reduction)	45–60% [28]	∼60% [29]	35.5–52.6% [30]	17–48% [31]

## 2. Mechanism of action

**Figure 1.**
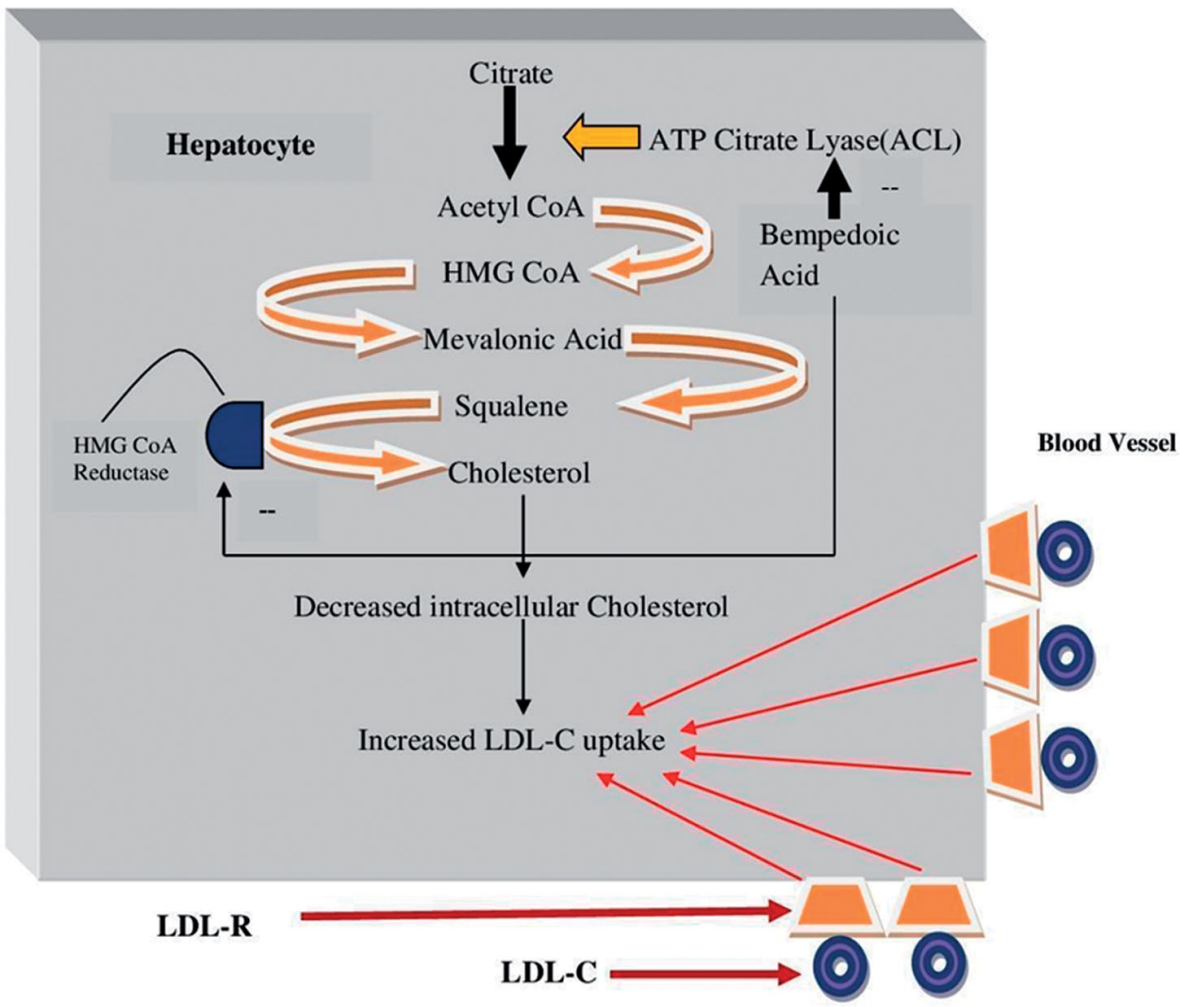
Mechanism of action of bempedoic acid.

Bempedoic acid (ETC-1002) (8-hydroxy-2,2,14,14-tetramethylpentadecanedioic acid) inhibits the activity of the enzyme ATP citrate lyase (ACLY). The ACLY enzyme plays a prominent role in lipid synthesis by converting citrate to acetyl CoA [13] (Figure 1). It functions in the same biosynthetic pathway as the enzyme HMG CoA Reductase (the target enzyme for statins), but lies upstream along the path. Due to the critical position of ACLY in the cholesterol pathway, multiple attempts have been made in the past to develop an inhibitor [14].

Bempedoic acid is once a day prodrug agent, taken orally with liver activation of the enzyme acyl-CoA synthetase [15]. The inhibition of ACLY by bempedoic acid decreases cholesterol synthesis, leading to upregulation of LDL receptors. Increased LDL receptor expression is associated with increased liver LDL uptake and therefore a decrease in serum LDL-C levels [16] (Figure 1). Bempedoic acid has high bioavailability due to its small size and rapid intestinal absorption. Statins and bempedoic acid, both exert their mechanism of action through machinery in the liver. However, the receptors utilized by the two drugs to enter the liver are different. This property of the bempedoic acid ensures that it does not competitively interrupt the uptake of statins in the liver [17,18]. The biosynthetic property of bempedoic acid makes it unique compared to statin, and the liver specific nature of the mechanism of action may be the reason behind lack of muscle related adverse effects seen with this prodrug [15].

In a preclinical study by Pinkosky et al., the authors demonstrated that besides inhibiting ACLY enzyme, the drug also activates the hepatic AMP kinase pathway [19]. AMP kinase inhibits the rate limiting enzymes of fatty acid pathway (acetyl CoA carboxylase) and cholesterol pathway (HMG-CoA reductase). Thus, bempedoic acid targets more than one pathway in lowering cholesterol levels [19].

Bempedoic acid affects its target enzymes only in the cells that can mediate its conversion from its inactive to active form. The enzyme that is responsible for this conversion is very long chain acyl CoAsynthetase-1 (ACSVL1) that is expressed in liver with minimal presence in the kidneys and skeletal muscles of humans. The lack of ACSVL1 in skeletal muscle has been hypothesised as the potential mechanism for the lower myotoxic effects of bempedoic acid as compared to statins [18]. The aggregate data from observational studies and randomized controlled trials suggest up to 29% of statin associated muscle side effects [20]. Pinkosky et al., demonstrated the mechanistic basis for ETC-1002 avoiding the myotoxic effects compared to statins due to the absence of ACSVL1 activity in skeletal muscles in both mice and humans [18]. Jadhav et al. recently presented findings of their study suggesting an equivalence in the LDL-C lowering capacity of statin and bempedoic acid combination, compared to high dose statin monotherapy, without the muscular side effects with the higher dose [21]. Additionally, bempedoic acid was not associated with prolongation of QT interval [22].

## 3. Preclinical studies

The function of bempedoic acid (ESP-55016 and then ETC-1002) in dyslipidemia was studied in multiple animal studies. It was initially recognised as ω-hydroxy-alkanedicarboxilyc acid (ESP 55016) with lipid lowering effect in female Zucker (fa/fa) rats. ESP 55016 showed reduction in serum non- high-density lipoprotein (HDL) cholesterol, triglyceride and non-esterified fatty acids. There was a dose dependent increase in serum HDL cholesterol levels and decrease in serum glucose and insulin levels. In rat hepatocytes, this drug suppressed lipid synthesis [23]. This mechanism of action led to the discovery of ETC-1002, a novel agent developed in treatment of cardio-metabolic diseases and dyslipidemia. This molecule affects AMP-activated protein kinase in a Ca (2+)/calmodulin-dependent kinase β-independent and liver kinase β 1-dependent manner. It forms a CoA thioester in the liver leading to reduction of cholesterol synthesis by inhibiting ACLY. One of the ACLY products, oxaloacetate, serves as a substrate of gluconeogenesis, possibly explaining the role of the drug in reduction of gluconeogenesis [19]. In addition, ETC-1002 reduced glucose production through FOXO1, phosphoenolpyruvate carboxykinase (PEPCK), and glucose-6-phosphatase (G6Pase) protein levels [20]. This suggested that risk of diabetes was reduced with ETC-1002 compared to statins [24]. Ference et al. performed a study with genetically constructed scores mimicking bempedoic acid and did not find association of diabetes in the ACLY score [25]. Samsoondar et al. showed the effect of ETC-1002 on diet-induced metabolic dysregulation using LDLr–/– mice. These mice on high fat, high cholesterol diet was given bempedoic acid (3-30mg/kg/day for 12 weeks) which showed reduction in cholesterol by 50% and triglycerides by 64% [26]. Reduction in hepatic and aortic inflammation was also noted, as measure by decrease in markers for inflammatory macrophages in mice treated with bempedoic acid [26,27].

## 4. Bempedoic acid ameliorates inflammation

High sensitivity C-Reactive protein (hsCRP) level is an independent predictor of ASCVD risk [28]. Multiple studies have shown that hsCRP level <2 mg/L with statin therapy or with LDL-C levels <70 mg/dL provided significant ASCVD risk reduction, while an elevated hsCRP level were associated with residual risk of ASCVD. This inflammatory marker is considered a risk modulator in the latest 2018 ACC/AHA/Multi-societies lipid guidelines [31].

Unlike PCSK9 inhibitors, in addition to its cholesterol lowering potential, bempedoic acid has been shown to possess strong systemic anti-inflammatory effects in animal studies [26,32]. Multiple animal studies indicated the possible potential benefit of bempedoic acid in reducing insulin resistance and cardiac complications of metabolic syndrome through the activation of AMP-activated protein kinase which plays an important role in lipid and glucose metabolism. Another proposed mechanism is the downregulation of inflammatory signalling pathways through AMP kinase activation [33]. Additionally, inhibition of ACLY by bempedoic acid has been demonstrated to reduce prostaglandin production and contribute to anti-inflammatory action [34] and may have some role in NASH as well [35]. Lauf et al., in their CLEAR Serenity study, showed that bempedoic acid was associated with a 24.3% reduction in hsCRP levels from baseline, reducing inflammation and possibly ASCVD risk [36].

## 5. Clinical development of bempedoic acid

### 5.1. Phase I trials

The drug’s safety and tolerability were well established with phase 1 clinical studies evaluating the effect of varying doses of the drug as compared with placebo. In a phase 1a study involving 53 subjects with mild dyslipidemia (average LDL-C 126.7 mg/dL and TG 100-350 mg/dL), participants were treated with increasing doses of the drug up to 120 mg daily. A 17% reduction in LDL-C level was observed with an average dose of 100 mg daily. The phase 1 b study enrolled 24 healthy subjects who received drug in dose increments (140, 180, and 220 mg daily). A reduction of 36% LDL-C levels was observed, compared to placebo (*p* < .0001). Finally, ETC-1002-011 study had 6 healthy male subjects to assess absorption, metabolism and excretion of single dose in urine and faeces [37]. No major adverse events were noted in phase I studies, despite renal impairment [38].

### 5.2. Phase II trials

Multiple phase 2 studies evaluated the lipid lowering efficacy of bempedoic acid as monotherapy or as an add on to statin therapy, ezetimibe or a combination of all three agents [24,39,40]. Doses of between 40 and 240 mg/day of bempedoic acid were used in these studies. The study population included hyperlipidemic patients with other cardiovascular risk factors including diabetes mellitus, hypertension and/or statin intolerance. The use of this drug as a single agent or in combination with other hypolipidemic agents was associated with significant improvements in lipid levels and inflammatory markers. The results of the studies are summarized in Table 3.

**Table 3.** Phase 2 and phase 3 clinical trials of bempedoic acid.

Name of the trial	Phase of study	Primary objective	Status/Result
Bempedoic Acid + Ezetimibe fixed-dose combination (FDC) study in patients with type 2 diabetes and elevated LDL-C	Phase 2 NCT03531905	Percent change of LDL-C from baseline to week 12, by FDC vs. Placebo and FDC vs. Ezetimibe.	Completed ETC-1002 lowered LDL-C and hsCRP levels in type 2 diabetes population without worsening glycemic control (34)
Evaluation of the efficacy and safety of bempedoic acid (ETC-1002) 180 mg, Ezetimibe 10 mg, and Atorvastatin 20 mg triplet therapy in patients with elevated LDL-C	Phase 2 NCT03051100	Percent change in low-density lipoprotein cholesterol (LDL-C) after 6 weeks	Completed In patients with or without statin intolerance, daily treatment with ETC-1002 lowered LDL-C with similar tolerable profile (15)
A study of pharmacokinetics, pharmacodynamics and safety of adding ETC-1002 to atorvastatin 80 mg	Phase 2 NCT02659397	Percent change in LDL-C from baseline to 4 weeks treatment. Percent change in hsCRP	Completed ETC-1002 lowered LDL-C with acceptable safety profile (37)
Evaluation of the efficacy and safety of bempedoic acid (ETC-1002) 180 mg, when added to PCSK9 inhibitor therapy	Phase 2 NCT03193047	Assess the 2-month efficacy of bempedoic acid 180 mg/day vs placebo in the reduction of LDL-C in patients on PCSK9i therapy	Completed Bempedoic acid showed less skeletal muscle side effects than seen with statins
Evaluation of long-term efficacy of bempedoic acid (ETC-1002) in patients with hyperlipidaemia at high cardiovascular risk (CLEAR Wisdom)	Phase 3 NCT02991118	Percent change in low-density lipoprotein cholesterol (LDL-C) after 12 weeks of therapy	Completed Addition of bempedoic acid to maximally tolerated statin resulted in significant LDL-C lowering over 12 weeks (39)
Evaluation of the efficacy and safety of bempedoic acid (ETC-1002) in patients with hyperlipidaemia and statin intolerant (CLEAR serenity)	Phase 3 NCT02988115	Percent change in low-density lipoprotein cholesterol (LDL-C) after 12 weeks of therapy	Completed Bempedoic acid found to be safe and effective option of lipid lowering in statin intolerant patients (23)
Evaluation of the efficacy and safety of bempedoic acid (ETC-1002) as add-on to Ezetimibe therapy in patients with elevated LDL-C (CLEAR tranquillity)	Phase 3 NCT03001076	Percent change in low-density lipoprotein cholesterol (LDL-C) after 12 weeks of therapy	Completed Bempedoic acid found to be a therapeutic option complementary to ezetimibe in statin intolerant patients who needs additional LDL lowering (40)
A study evaluating the safety and efficacy of bempedoic acid plus ezetimibe fixed-dose combination compared to bempedoic acid, ezetimibe, and placebo in patients treated with maximally tolerated statin therapy	Phase 3 NCT03337308	Percent change in low-density lipoprotein cholesterol (LDL-C) after 12 weeks of therapy	Completed Fixed dose combination significantly lowered LDL-C compared to placebo or other monotherapies with favourable safety profile (41)
Evaluation of long-term safety and tolerability of ETC-1002 in high-risk patients with hyperlipidaemia and high CV risk (CLEAR HARMONY)	Phase 3 NCT02666664	Incidence of treatment related adverse effects	Completed Bempedoic acid added to maximally tolerated statin therapy did not lead to a higher incidence of overall adverse events than placebo and led to significantly lower LDL cholesterol levels (42)
Assessment of the long-term safety and efficacy of bempedoic acid (CLEAR Harmony OLE)cl	Phase 3 NCT03067441	Incidence of adverse events in patients with high cardiovascular risk and elevated LDL cholesterol	Active Not yet published
Evaluation of major cardiovascular events in patients with, or at high risk for, cardiovascular disease who are statin intolerant treated with bempedoic acid (ETC-1002) or Placebo (CLEAR OUTCOMES)	Phase 3 NCT02993406	Time to first occurrence of MACE	Recruiting Not yet published (45)

**Table 4.** Key highlights of bempedoic acid.

February 2020, USA FDA approved bempedoic acid as a single agent and bempedoic acid with ezetimibe combination for adults with heterozygous familial hypercholesterolaemia or established atherosclerotic cardiovascular disease. Phase III trials showed its efficacy in lowering LDL-C It reduces hs-CRP level besides reduction of LDL-C Weight neutral, does not affect blood pressure or glucose metabolism Need future trials to look for this simultaneous LDL-C and hs-CRP lowering effects translating into CV mortality benefits

Another phase-II trial (NCT02659397) published by Lalwani et al. studied the effect of adding bempedoic acid to stable high intensity atorvastatin therapy in patients with hypercholesterolaemia. Patients received 80 mg atorvastatin for 4 weeks followed by randomization in ratio of 2:1 to receive bempedoic acid 180 mg daily or placebo for 4 weeks. The mean LDL level was lowered by 22% with bempedoic acid compared to baseline (*p* = .003). The addition of a stable dose of bempedoic acid to high intensity atorvastatin 80 mg reduced the non HDL cholesterol (-13%; *p* = .015), apolipoprotein B (−15%; *p* = .004), and high-sensitivity C-reactive protein (−44%; *p* = .002) significantly, with no significant alteration of the concentration of atorvastatin or its major metabolite ortho-hydroxy atorvastatin [39].

### 5.3. Phase III trials

The CLEAR trial series are all phase III randomised clinical trials focussed on evaluating the efficacy of bempedoic acid. The initial four trials (CLEAR Tranquillity, CLEAR Serenity, CLEAR Wisdom and CLEAR Harmony) have evaluated the LDL-C lowering efficacy of the drug while the CLEAR OUTCOME trial is an ongoing trial focussed on evaluating the cardiovascular outcomes with the drug.

The safety and efficacy of bempedoic acid 180 mg daily as an add on therapy to ezetimibe 10 mg daily in patients with a history of statin intolerance and those who had elevated LDL-C (LDL-*C* ≥ 100 mg/dL) on their current therapy was studied in CLEAR Tranquillity. The study subjects were randomized to oral bempedoic acid 180 mg or placebo once daily for 12 weeks. Bempedoic acid yielded an additional 28.5% LDL-C lowering compared with placebo (*p* < .001; −23.5% bempedoic acid, +5.0% placebo). A significant improvement was also seen in other lipid and lipoprotein parameters, including non-HDL-C, total cholesterol and apoB level. A median hsCRP reduction of 33% was noted at week 12 (*p* < .001). Besides showing lipid lowering efficacy, bempedoic acid was also well tolerated in the study arm. Adverse events of the drug were included increase in serum uric acid level (7.7% in bempedoic acid group compared to 2.3% in placebo group) and headache (4.4% in bempedoic acid and 3.4% in placebo group). Muscle-related adverse effects including muscular weakness, myalgias and rhabdomyolysis were similar in both the study and the placebo arms (3.3% in bempedoic acid and 3.4% placebo). The most important limitation of the trial was the short duration of 12 weeks over which outcomes were measured [41]. Subsequently, the CLEAR Harmony trial was a longer (24 weeks) study involving patients with either ASCVD, heterogeneous FH or both. In this trial, 2230 patients who were on guideline directed statin therapy were randomized into two groups (bempedoic acid versus placebo) and the baseline mean LDL-C was 103 ± 29.4 mg per decilitre. The incidence of serious adverse events was comparable in both the groups (14.5% in bempedoic acid group and 14.0% in the placebo group). Similar to the results of the previous trials, myalgia and other muscle related serious effects were similar in both groups while gout occurred more often in the bempedoic acid group (1.2%) compared to the placebo group (0.3%). Bempedoic acid reduced LDL cholesterol level by 18.1% (95% CI: −20.0 to −16.1, *p* < .001) at week 12 which persisted at week 24 (16.1%, 95% CI: −18.4 to −14, *p* < .001) when compared to the placebo. There was a lower incidence of diabetes and worsening of the disease in bempedoic acid group (3.3% in bempedoic acid group compared to 5.4% in placebo group, *p* = .02), but due to low event rate the study was under-powered to specifically study this effect [42].

While the above two trials evaluated the efficacy of bempedoic acid as an add-on therapy in patient on maximal statin therapy, the CLEAR Serenity trial studied 345 patients with hypercholesterolaemia who were intolerant to at least two statins (one of them at the lowest available dose). These patients were followed for over 24 weeks to the primary end point of percentage change in LDL-C level from baseline with bempedoic acid 180 mg. At week 12, bempedoic acid significantly lowered LDL-C level from baseline (95% CI: −25.1 to −17.7%, *p* < .001). There was also a significant reduction in hsCRP compared to baseline in the bempedoic acid group. The adverse event rate was higher in bempedoic acid group 64.1% as compared to the 56.8% in placebo group. The common side effects included musculoskeletal and connective tissue disorders (22.2 versus 25.2% in bempedoic acid and placebo groups, respectively). This trial expanded the evidence regarding the use of bempedoic acid in statin intolerant population in long term period [36].

A reduction in LDL-C levels among ASCVD or heterogenous FH patients on maximum tolerated statin therapy using bempedoic acid was first investigated in the CLEAR Wisdom trial. It was a randomized controlled trial with 779 patients to the drug group (180 mg bempedoic acid) or placebo once daily. The baseline LDL-C was 120 mg/dL with a follow up of 52 weeks. The primary outcome was a significant change in the LDL-C levels from baseline to week 52 (−15.4 vs 2.4% in bempedoic acid group compared to placebo). The study was underpowered, making it difficult to interpret clinical outcomes [43]. Banach et al. performed a meta-analysis of these 4 trials and confirmed a statistically significant reduction of LDL-C with bempedoic acid, when compared to the 1.5% reduction in the placebo group at week 12 (−24.5%; 95% CI, −27.8% to −21.1%; *p*<.001). The reduction in the levels of LDL-C levels persisted at week 52 in patients with ASCVD, HeFH or both receiving a maximally tolerated statin (−12.7% difference between bempedoic acid and placebo group) [44]. Similar results were confirmed in other meta-analysis, with no significant increase in adverse effects noted with bempedoic acid [45,46].

While all the trials have shown consistent reduction in LDL-C levels with bempedoic acid, two recently published systematic analyses have also shown a lower incidence of new onset and worsening of diabetes mellitus [47–50]. This has the additional potential in expanding the use of the drug in diabetes dyslipidemia and could be explained by the modest reduction in triglyceride levels mitigated by bempedoic acid. In a recently published meta-analysis of the four RCTs on bempedoic acid, it was shown to reduce the levels of hsCRP (−23.4% (CI 95% −32.6 to −14.2)]; *p* < .05; *I*^2^ = 69%) [51,52]. The latest trial in the series (CLEAR Outcomes trial) is aimed at studying the cardiovascular outcomes with bempedoic acid 180 mg daily. It is the largest trial randomizing 14,014 high cardiovascular disease risk statin intolerant patients with a follow up period of 36–42 months [53].

The majority of the phase-3 trials compared bempedoic acid alone with either placebo or statins. One recently published phase-3 trial compared the safety and efficacy of fixed dose combination (FDC) of bempedoic acid 180 mg along with ezetimibe 10 mg in high-risk patients on maximum tolerated statin [54]. In this trial, 382 patients were randomized into 4 groups in ratio 2:2:2:1 to oral bempedoic acid 180 mg plus ezetimibe 10 mg daily combination, bempedoic acid 180 mg daily, ezetimibe 10 mg daily or placebo for 12 weeks. Significant reduction in percentage change in LDL level at end of week 12 was seen in FDC group (−36.2%) in comparison to bempedoic acid as single agent −17.2%; *p* < .001), ezetimibe only (−23.2%; *p* < .001) and placebo (1.8%; *p* < .001). The FDC bempedoic acid and ezetimibe also lowered the hs-CRP (35.1%) and other lipid levels including non-HDL-cholesterol, total cholesterol and apolipoprotein B. The adverse events were frequently reported in FDC and bempedoic acid groups compared to ezetimibe alone or placebo groups. Among the FDC group, the most common side effects were constipation, muscle spasms, fatigue and blood uric acid increase. In a subgroup analysis of patients who were not receiving statin due to intolerance, FDC reduced LDL-C by 38.8% and subgroup analysis of patients on high-intensity statin, FDC reduced LDL-C by 38.9% [54]. These interesting findings support the use of bempedoic acid and ezetimibe combination in lowering LDL, even in population not on a medication from the statin class of drugs [54]. In a pooled analysis of 388 patients, Bhagavathula et al. also noted a significant reduction of LDL-C (− 29.14%, 95% CI −39.52 to −18.76; *p*<.001) and hsCRP (− 30.48%, 95% CI −44.69 to −16.28; *p* = .04) with the combination of bempedoic acid and ezetimibe in this patient population, as compared to the control group, after 12 weeks [55]. In a smaller trial enrolling 59 patients on background therapy with PCSK9 inhibitors, the addition of bempedoic acid showed a further reduction of LDL-C by 30.3% after 2 months of therapy, when compared with placebo [56].

## 6. Conclusion and future direction

For many decades, statins were the only hypolipidemic drugs available with a proven cardiovascular mortality benefit. However, many patients either could not achieve optimal lipid goals on maximally tolerated statin doses or experienced statin intolerance. The last 5 years have seen significant advances in the development and evidence basis for novel therapies for hyperlipidaemia and cardiovascular diseases, with many new molecules receiving approval in both North America and Europe. There have been multiple updated guidelines from the ACC/AHA and ESA/ESC to keep pace with this advancement. Parallel to this surge in development of new classes of drugs, recent clinical trials have shown improvement in cardiovascular mortality with additional reduction in the LDL-C levels. Thus, the target LDL-C goal for primary and secondary prevention for “very high-risk” and “high-risk” ASCVD patients has progressively become lower over the decades. Drugs including ezetimibe, PCSK9 inhibitors, bempedoic acid are now available for clinicians to target these LDL-C goals, and pick the most optimized combination with maximum cost effectiveness of hypolipidemic drugs for their patients [57]. Keeping focus on this patient-centric approach, the recently approved bempedoic acid-ezetimibe combination could be tried prior to attempting PCSK9 inhibitors, which have certain limitations including injectable preparation and higher costs. The fixed drug combination of bempedoic acid and ezetimibe has shown LDL-C reduction comparable to PCSK9 inhibitors in clinical trials. In February 2020, US FDA (Table 4) approved oral bempedoic acid, once a day medication, for use in heterozygous hypercholesterolaemia or established ASCVD patients, who need further LDL-C lowering in addition to dietary modifications and maximally tolerated statin. The once daily oral regimen and the favourable adverse effect profile makes bempedoic acid an appealing alternative. However, one major limitation of use of bempedoic acid includes lack of CVD outcome data, which will be addressed in future clinical trials. A recent study identified insurance approvals and cost as additional barriers which will also require to be addressed to promote in real world clinical practice [58].

## Data Availability

The authors confirm that the data supporting the findings of this study are available within the article [and/or] its supplementary materials.
